# Removal of Aqueous Antimony and Arsenic by Iron-Loaded Coal Gasification Slag Composite

**DOI:** 10.3390/toxics12060440

**Published:** 2024-06-19

**Authors:** Zheng Leng, Changzhi Zhou, Hong Hou, Junhuan Wang

**Affiliations:** State Key Laboratory of Environmental Criteria and Risk Assessment, Chinese Research Academy of Environmental Sciences, Beijing 100012, China; lengchongxin@outlook.com (Z.L.); zhouchangzhi1018@163.com (C.Z.)

**Keywords:** iron-loaded coal gasification slag, arsenic, antimony, FeOOH, adsorption

## Abstract

The adsorption of Sb(V) and As(V) onto iron-loaded gasification slag composite material (Fe-GFS), as well as the possible mechanisms, was investigated. Batch experiments showed that in a single system, Fe-GFS sorbed As(V) to a greater extent than Sb(V) with the maximum adsorption capacity (pH 3.0) of 34.99 mg/g (0.47 mmol/g), while that of Sb(V) was 27.61 mg/g (0.23 mmol/g). In the composite system, the presence of low concentrations of Sb(V) reduced the adsorption efficiency of Fe-GFS for As(V), while the presence of high concentrations of Sb(V) actually promoted the adsorption of As(V). The presence of As(V) consistently inhibited the adsorption of Sb(V) by Fe-GFS. Compared to Fe-GFS, new peaks appeared in the FTIR spectra after adsorption, indicating the presence of Sb-O and As-O bonds on the surface after adsorption. XPS results showed that the adsorption of As(V) and Sb(V) led to a decrease in Fe-OH bonds, with a more significant decrease in Fe-OH bonds observed after the adsorption of As(V), indicating a stronger affinity of Fe-GFS for As(V) compared to Sb(V). Our results suggest that Fe-GFS is an efficient adsorbent with great potential for applications in water containing As(V) and Sb(V).

## 1. Introduction

China is the world’s largest antimony reserve and producer [1], with numerous antimony mining areas concentrated in regions such as Hunan, Guizhou, and Guangxi. During the process of antimony mining, a large amount of soluble antimony salts is dissolved in the discharged beneficiation wastewater. Additionally, antimony can form coexisting ore deposits with arsenic in the form of sulfide [2] and, as a result, arsenic often synchronously enters the wastewater with the release of antimony, causing complex pollution of antimony and arsenic, thereby posing a huge threat to the surrounding environment of the mining area. Studies have shown that the concentrations of As and Sb in the water bodies around the Xikuangshan were 190 μg/L and 3700 μg/L, respectively [3]; in the vicinity of antimony mines in the Slovak Carpathians region, high concentrations of antimony and arsenic, reaching 9300 μg/L and 2150 μg/L, respectively, were found in the water [4]; in the Hillgrove area of Australia, the oxidation of ores such as stibnite resulted in a large amount of As and Sb entering nearby rivers, with concentrations reaching 300 μg/L and 1800 μg/L, respectively [5]. As two highly toxic elements, antimony and arsenic can cause a decrease in enzyme activity and lead to various diseases, including cardiovascular diseases, when they are absorbed by the human body [6,7]. After being absorbed by aquatic organisms or crops, aqueous arsenic and antimony can further harm human health through the food chain [8]. Therefore, the removal of aqueous arsenic and antimony combined pollution has become an urgent and important issue in the field of environmental protection.

The main methods for removing aqueous antimony and arsenic include adsorption, membrane filtration, electrocoagulation, and ion exchange, among others. Among them, adsorption is simple, efficient, cost-effective, and adaptable, making it the most widely used technology for removing aqueous antimony and arsenic [9]. Adsorption is a process that involves physical or chemical adsorption based on the electrostatic interactions or chemical bonds between high-surface-area insoluble solids and adsorbates [10]. There are various types of adsorption materials that have been utilized to remove aqueous antimony and arsenic, such as clay minerals, biochar, metal oxides, industrial by-products, and so forth. Among them, industrial by-product adsorption materials, with abundant reserves, low cost, easy accessibility, and environmental friendliness, have attracted wide attention.

Coal gasification slag is an industrial by-product formed from inorganic minerals along with residual carbon particles during the gasification process. With coal as the primary energy source in China’s energy structure, the production of gasification slag has been increasing as gasification scales up. Currently, the main methods for handling coal gasification slag are stacking and landfilling, leading to environmental pollution and wastage of land resources, thus making the treatment of coal gasification slag urgent. Comprising residual carbon, SiO_2_, Al_2_O_3_, CaO, and Fe_2_O_3_, the composition of coal gasification slag varies to a certain extent depending on the properties of the original coal and the feeding method. In addition, coal gasification slag has a large specific surface area, well-developed porosity, and is rich in carbon and silicon resources [11]. After certain treatments, it can be used as an adsorbent to treat pollutants in water. Studies have shown that after a series of modifications, gasification slag exhibits excellent adsorption effects on methylene blue [12], ${\mathrm{NH}}_{4}^{+}$, and ${\mathrm{PO}}_{4}^{3-}$ [13]. Bao et al. [14] modified gasification slag through grinding and hydrofluoric acid treatment, and the modified material achieved maximum adsorption capacities of 0.54 mmol/g for Pb^2+^, 0.63 mmol/g for Cu^2+^, and 0.28 mmol/g for Cd^2+^. Deng et al. [15] prepared a Zr-loaded gasification slag adsorbent, and under optimized conditions, the Zr-loaded gasification slag exhibited an adsorption capacity of 6.82 mg/g for Cr^6+^. However, most studies have focused on the adsorption of organic pollutants and heavy metals from water by coal gasification slag-based materials, with limited research on the adsorption of metalloids like arsenic and antimony.

Studies have shown that the adsorption of aqueous antimony and arsenic mainly depends on electrostatic attraction, ion exchange, and complexation. In a binary system of antimony and arsenic, there will be mutual influence between each other. Qi et al. [16] found in a binary system that As(V) inhibits the adsorption of Sb(V) by ferrihydrite, while the presence of Sb(V) has no effect on the adsorption of As(V). On the other hand, Wu et al. [17] found that the presence of Sb(V) promotes the adsorption of As(V) by ferrihydrite at neutral pH. These studies examining the mutual interactions between Sb(V) and As(V) in binary systems often overlook the potential impact of differences in Sb(V) and As(V) concentrations, which is highly common in environmental settings. Therefore, this study intends to use iron-modified coal gasification slag as the base material to investigate its adsorption capacity for Sb(V) and As(V). The study aims to explore how varying concentrations of Sb(V) and As(V) may affect each other in a binary system and ultimately investigate the potential of modified coal gasification slag materials as adsorbents for aqueous Sb(V) and As(V).

## 2. Materials and Methods

### 2.1. Materials

The coal gasification slag used in the experiment was produced by Hexinheyuan Environmental Protection Technology Co., Ltd., Yulin City, China. The stock solutions of As(V) and Sb(V) were prepared from the dissolution of Na_2_HAsO_4_·7H_2_O (>98%, Sigma-Aldrich, Shanghai, China) and KSbO_6_H_6_ (99%, Macklin, Shanghai, China), respectively. KOH and FeCl_3_·6H_2_O (AR grade, Macklin, Shanghai, China) were used for the synthesis of Fe-GFS. The desired pH for the experiment is adjusted using 0.1 M HCl or NaOH. All reagents are prepared using deionized water (Master-S15, HHitech, Shanghai, China).

The preparation of the Fe-GFS followed the method by Zhou [18]: coal gasification slag and KOH were mixed at a mass ratio of 0.8:1. The mixture was then placed in a tubular furnace, heated at a rate of 10 °C per minute to 800 °C under a nitrogen flow rate of 100 cm^3^/min, and maintained for 1.5 h before natural cooling. The cooled material was slowly added to a 0.2 mol/L FeCl_3_ solution with continuous stirring until the suspension reached a pH of 7. The suspension was centrifuged at 4000 rpm for 5 min to remove the supernatant. The precipitate was collected and subjected to freeze-drying in a vacuum freeze-dryer. The material obtained after freeze-drying was named Fe-GFS.

### 2.2. Characterization of Fe-GFS

Observation of the surface microstructure and morphology of Fe-GFS was conducted using scanning electron microscopy (SEM; ZEISS GeminiSEM 300, Beijing Precise instrument Co., Ltd., Beijing, China), while analysis of the elemental composition of Fe-GFS was performed using energy-dispersive X-ray spectroscopy (EDS; Spectra Ultra, Therom Ficher Scientific, Beijing, China). The specific surface area of Fe-GFS was calculated using the Brunauer–Emmett–Teller (BET) method, and the pore size distribution of Fe-GFS was determined using the Barrett–Joyner–Halenda (BJH) method. The functional groups on the surface of Fe-GFS were identified using Fourier transform infrared spectroscopy (FTIR; Nicolet 6700, Therom Ficher Scientific, Beijing, China). The surface elemental composition and chemical states of Fe-GFS were analyzed by X-ray photoelectron spectrometry (XPS; ESCALAB 250Xi, Thermo Ficher Scientific, Beijing, China).

### 2.3. Adsorption Experiment in Aqueous System

#### 2.3.1. Adsorption Isotherms

A series of As(V) solutions with concentrations ranging from 5 to 100 mg/L were prepared for isothermal adsorption experiments. The initial pH of each solution was 3. Then, 0.02 g of Fe-GFS was added to 25 mL of As(V) solution, and the mixture was shaken for 24 h at 25 °C, followed by centrifugation at 4000 rpm for 5 min. The supernatant was extracted and filtered using a 0.22 μm filter membrane, and the As(V) content in the filtrate was determined. Each treatment was repeated three times. The experimental data were fitted using the Langmuir and Freundlich models. The same procedure was repeated for Sb(V).

#### 2.3.2. Adsorption Kinetics

The As(V) solutions with an initial concentration of 20 mg/L were prepared to study the adsorption kinetics. The initial pH of the solutions was 3. Then, 0.02 g of Fe-GFS was added into 25 mL As(V) solution, and the mixture was shaken at 25 °C for 5, 10, 30, 60, 120, 240, 480, 720, and 1440 min, respectively, followed by centrifugation at 4000 rpm for 5 min. The supernatant was extracted and filtered through a 0.22 μm filter membrane, and the As(V) content in the filtrate was determined. Each treatment was repeated three times. The pseudo-first-order model and pseudo-second-order kinetics model were adopted to describe the adsorption rates. The same procedure was repeated for Sb(V).

#### 2.3.3. Adsorption Experiments at Different pH Values

The initial concentration of As(V) solutions at 20 mg/L was adjusted to pH values of 3, 4, 5, 7, 9, and 11, respectively. Then, 0.02 g of Fe-GFS was added to 25 mL of As(V) solution, and the mixture was shaken for 24 h at 25 °C, followed by centrifugation at 4000 rpm for 5 min. The supernatant was extracted and filtered using a 0.22 μm filter membrane, and the As(V) content in the filtrate was determined. Each treatment was repeated three times. The experimental data were fitted using the Langmuir and Freundlich models. The same procedure was repeated for Sb(V).

#### 2.3.4. Binary Adsorption of As and Sb

The binary adsorption of As(V) and Sb(V) was conducted at pH 6.87 for 24 h. A total of nine combinations were set: (a) As100Sb0; (b) As100Sb10; (c) As100Sb20; (d) As100Sb50; (e) As100Sb100; (f) As0Sb100; (g) As10Sb100; (h) As20Sb100; (i) As50Sb100 (Note: As100Sb10 represents the binary mixed solution with 100 mg/L As and 10 mg/L Sb, and the same for the other combinations). The adsorption conditions were consistent with those of isotherm adsorption experiments. After adsorption, the solutions of groups a, e, and f were filtered, and the filtered material was allowed to dry and used for characterization.

### 2.4. Data Analysis

The data were expressed as mean ± standard deviation of three replicates. Statistical analysis was performed using IBM SPSS Statistics25 software. One-way analysis of variance (ANOVA) was used to evaluate the significant differences among groups, and *p* < 0.05 was statistically significant.

## 3. Results and Discussion

### 3.1. Adsorption Isotherms

**Figure 1:**
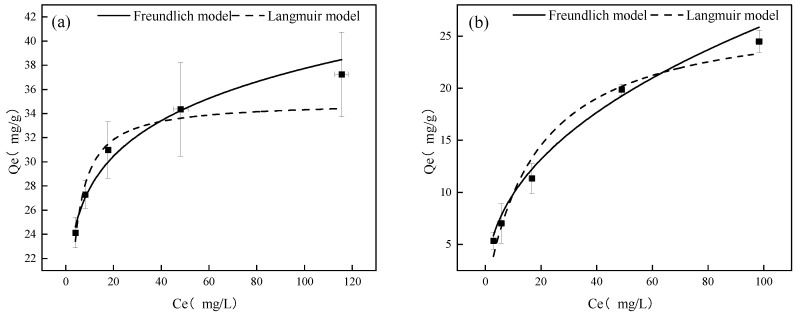
The adsorption isotherms of As(V) (**a**) and Sb(V) (**b**) on Fe-GFS.

To investigate the adsorption mechanism of As(V) and Sb(V) by Fe-GFS, isothermal adsorption experiments were conducted at 25 °C, and the experimental results were fitted using the Langmuir and Freundlich models. As shown in Figure 1, the equilibrium adsorption capacities of Fe-GFS for As(V) and Sb(V) increased with increasing equilibrium concentrations of As(V) and Sb(V). When the equilibrium concentration of As(V)/Sb(V) was less than 40 mg/L, the adsorption capacity increased rapidly with the increase in As(V)/Sb(V) concentration in the solution. However, when the equilibrium concentration of As(V)/Sb(V) exceeded 40 mg/L, the rate of increase in adsorption capacity slowed down. Both models could fit the adsorption of Fe-GFS on As(V) and Sb(V) well; however, from the fitting parameters in Table 1, it can be seen that the Freundlich model had better fitting coefficients of 0.966 and 0.984 for As(V) and Sb(V), respectively, compared to the Langmuir model with coefficients of 0.896 and 0.972, respectively. The Langmuir model mainly describes monolayer adsorption on a uniform surface [19], while the Freundlich model assumes multilayer adsorption on a non-uniform surface [20], indicating that the adsorption of Fe-GFS on As(V) and Sb(V) is a multilayer adsorption on a non-uniform surface. The Freundlich adsorption isotherm parameters 1/n were 0.13 (As(V)) and 0.42 (Sb(V)), both between 0 and 1, indicating a strong adsorption capacity of Fe-GFS for As(V) and Sb(V). According to the Langmuir model fitting under experimental conditions, the maximum adsorption capacities of Fe-GFS for As(V) and Sb(V) were found to be 34.99 mg/g (0.47 mmol/g) and 27.61 mg/g (0.23 mmol/g), respectively, suggesting that Fe-GFS had a better adsorption effect on As(V) than Sb(V). A comparison with data from other adsorbent materials (Table 2) reveals that Fe-GFS exhibits strong adsorption capacities for both As(V) and Sb(V).

### 3.2. Adsorption Kinetics

**Figure 2:**
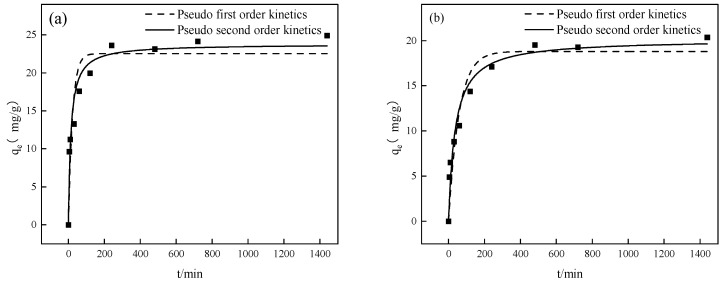
Adsorption kinetics of As(V) (**a**) and Sb(V) (**b**) on Fe-GFS.

To investigate the adsorption performance of Fe-GFS on As(V) and Sb(V), kinetic adsorption experiments were conducted, and the experimental results were fitted using pseudo-first-order and pseudo-second-order kinetic models. As shown in Figure 2, it can be observed that in the initial stage of the adsorption process, the adsorption of As(V) and Sb(V) by Fe-GFS rapidly increased with the extension of adsorption time, followed by a slowing down of the adsorption rate, and the fitted curves tended to stabilize. The adsorption of As(V) and Sb(V) both reached equilibrium after approximately 200 min. The parameters for the adsorption kinetics simulation are shown in Table 3. In the adsorption process of Fe-GFS on As(V) and Sb(V), the correlation coefficients of the pseudo-first-order kinetic model were 0.8593 and 0.9078, both lower than those of the pseudo-second-order kinetic model (0.9433 for As(V) and 0.9574 for Sb(V)). It can be seen that the pseudo-second-order kinetic model is more suitable for describing the dynamic adsorption changes of Fe-GFS on As(V) and Sb(V), indicating that the step limiting the adsorption rate may be the reaction. Fe-GFS rapidly adsorbs most of the As(V)/Sb(V) ions in the solution at the initial stage, and then the adsorption rate slows down. This may be because, in the early stages of the reaction, there are many unoccupied adsorption sites on the surface of Fe-GFS, leading to a short adsorption time and a fast adsorption rate. As many active sites are occupied by the adsorbate, complexation becomes the main driving force for adsorption, gradually reaching adsorption equilibrium.

### 3.3. Effect of pH on As(V) and Sb(V) Adsorption

**Figure 3:**
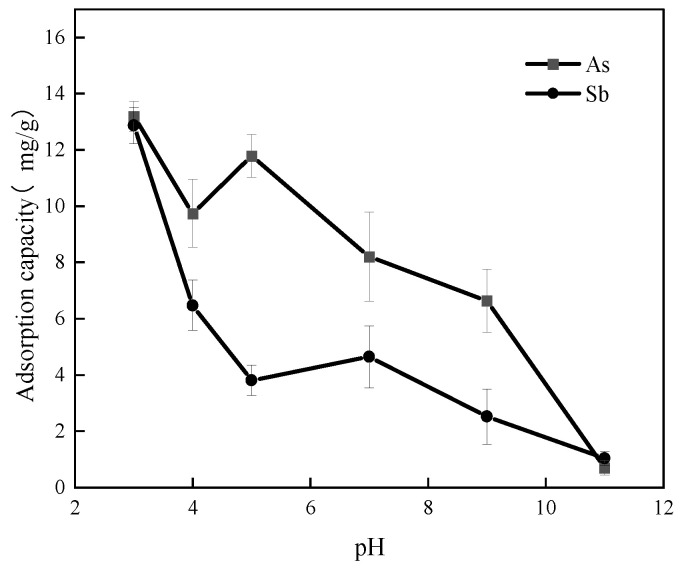
Effect of different solution pH on the adsorption of As(V) and Sb(V) by Fe-GFS.

To investigate the effect of initial solution pH on the adsorption of As(V) and Sb(V) by Fe-GFS, adsorption experiments were conducted at various pH. The results are shown in Figure 3. At pH 3, the maximum adsorption capacities of As(V) and Sb(V) by the material were achieved, reaching 13.18 mg/g and 12.87 mg/g, respectively. The adsorption capacities gradually decreased with increasing pH, and at pH 11, the adsorption capacities of As(V) and Sb(V) by Fe-GFS were 0.71 mg/g and 1.04 mg/g, respectively. In aqueous environments with pH greater than 3, Sb(V) primarily exists in the form of $\mathrm{Sb}{\left(\mathrm{OH}\right)}_{6}^{-}$ [24,25]. At pH 3, the surface protonation of Fe-GFS is strong, promoting the adsorption of the anionic $\mathrm{Sb}{\left(\mathrm{OH}\right)}_{6}^{-}$ through electrostatic attraction. As the pH increases, deprotonation occurs on the surface of Fe-GFS, resulting in an increase in surface negative charge and subsequent electrostatic repulsion, leading to a gradual decrease in the adsorption of $\mathrm{Sb}{\left(\mathrm{OH}\right)}_{6}^{-}$ by Fe-GFS. Additionally, the increase in OH^−^ concentration in the solution competes for adsorption sites on the material surface, further reducing the adsorption capacity of Fe-GFS for $\mathrm{Sb}{\left(\mathrm{OH}\right)}_{6}^{-}$. Similarly, for As(V), in aqueous environments with pH greater than 3, As(V) exists in forms such as $H_{2}{\mathrm{AsO}}_{4}^{-}$, ${\mathrm{HAsO}}_{4}^{2-}$, and ${\mathrm{AsO}}_{4}^{3-}$ [26]. Therefore, as the pH of the solution increases, electrostatic repulsion and OH^−^ gradually occupy active sites, leading to a gradual decrease in the adsorption capacity of Fe-GFS for arsenate ions. The solution pH affects the surface charge of Fe-GFS and the existing forms of As(V) and Sb(V), thereby influencing the adsorption capacity of Fe-GFS for As(V) and Sb(V). This indicates that Fe-GFS can adsorb aqueous As(V) and Sb(V) through electrostatic interactions.

### 3.4. Adsorption in Binary Systems

To investigate the effect of Sb(V) and As(V) on each other, adsorption experiments were carried out in binary systems with different antimony and arsenic ratios, and the results are shown in Table 4. In the binary solution of As(V) and Sb(V), maintaining an initial mass concentration of Sb(V) at 100 mg/L while changing the As(V) concentration, the adsorption capacity of Fe-GFS for As(V) increased from 1.95 mg/g to 13.58 mg/g as the As(V) concentration rose from 0 to 100 mg/L. Simultaneously, the adsorption capacity of Sb(V) decreased from 14.89 mg/g to 3.77 mg/g. This indicates a significant competitive effect between As(V) and Sb(V) in the binary system, where As(V) can inhibit the adsorption of Sb(V) by Fe-GFS.

Maintaining an initial mass concentration of As(V) at 100 mg/L, as the Sb(V) concentration increased from 0 to 100 mg/L, the adsorption capacity of Fe-GFS for Sb(V) was essentially 0, further demonstrating the competitive effect between As(V) and Sb(V). When the Sb(V) concentration was 0, the adsorption capacity of Fe-GFS for As(V) was 12.21 mg/g. As the Sb(V) concentration increased to 10 and 20 mg/L, the adsorption capacities for As(V) were 10.67 and 11.29 mg/g, respectively, showing a decrease compared to individual adsorption of As(V). When the Sb(V) concentration increased to 50 and 100 mg/L, the adsorption capacities of Fe-GFS for As(V) increased to 13.20 and 13.58 mg/g, respectively. In the binary system of As(V) and Sb(V), the presence of low levels of Sb(V) inhibits the adsorption of As(V) by Fe-GFS, while the presence of high concentrations of Sb(V) actually promotes the adsorption of As(V). This may be due to the ability of high Sb(V) concentrations to form As-O-Sb bonds on the surface of Fe-GFS, exhibiting a certain synergistic effect [27].

### 3.5. Adsorption Mechanism of As(V)/Sb(V) on Fe-GFS

**Figure 4:**
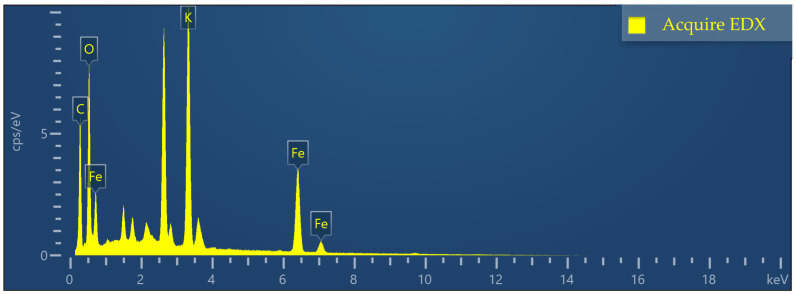
EDS analysis of Fe-GFS.

**Figure 5:**
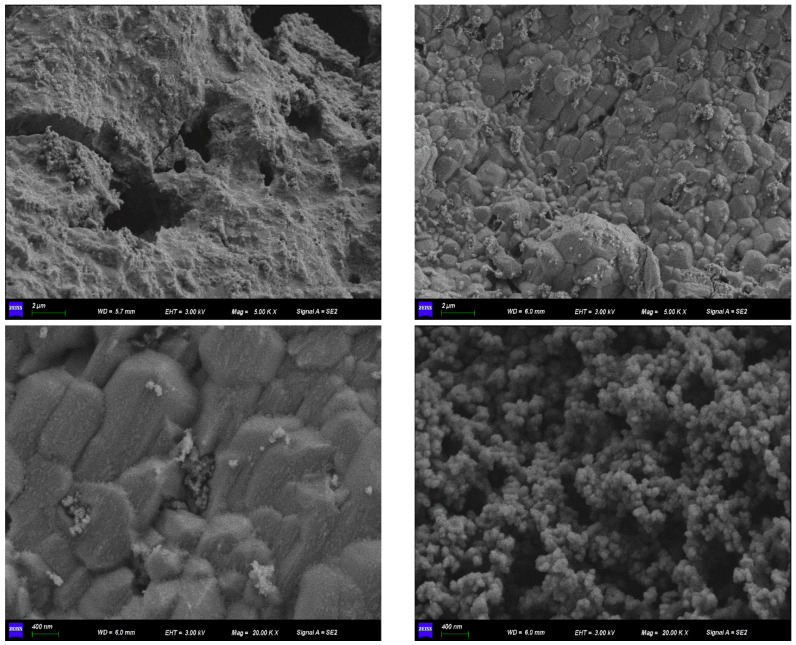
SEM image of Fe-GFS.

To explain the adsorption mechanism of Fe-GFS for As and Sb, the composition, morphology, and structure of the material before and after adsorption were characterized. EDS analysis in Figure 4 shows that the surface elemental composition of Fe-GFS was C, O, Fe, and K, with atomic percentages of C (48.06%), O (35.78%), Fe (8.26%), and K (7.90%), respectively, preliminarily proving that Fe was successfully loaded onto the material surface. The scanning electron microscope image of Fe-GFS, as shown in Figure 5, revealed a rough and porous surface with some collapsed pores, possibly due to local etching of the material during the impregnation process with acidic FeCl_3_ [28]. Additionally, many irregular particles were uniformly attached to the surface of Fe-GFS, similar to β-FeOOH obtained by Xu et al. [29], displaying significant aggregation forming irregular clusters, likely stemming from the partial phase transformation of β-FeOOH to α-FeOOH leading to simultaneous aggregation [30].

**Figure 6:**
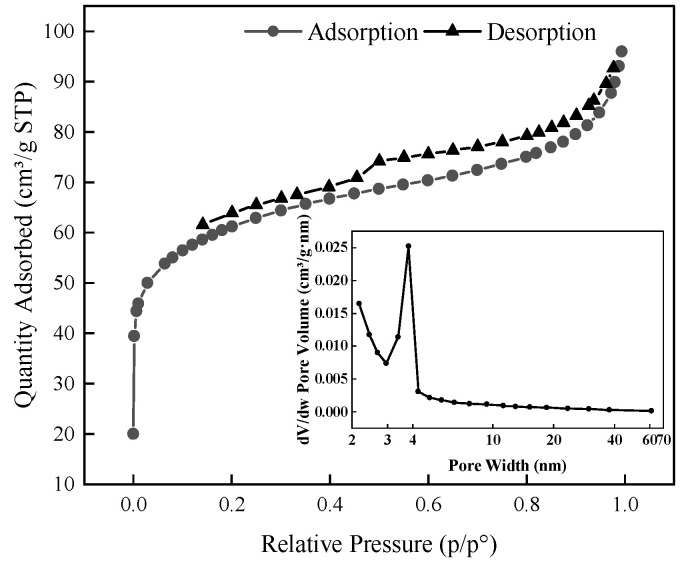
BET adsorption isotherm of Fe-GFS.

The N_2_ adsorption/desorption isotherm of Fe-GFS, shown in Figure 6, indicated that according to the IUPAC classification, the BET adsorption isotherm of Fe-GFS closely resembled Type IV, suggesting that Fe-GFS possessed mesoporous properties. The hysteresis loop conformed to Type H4, which is typically associated with narrow slit-like pores, including pore regions of micropores [31], indicating that the surface of Fe-GFS still contained some micropores that were not closed, possibly due to the deformations caused by adsorption or pore filling in the coal gasification slag material, which is not a rigid structure [32]. It could also be due to the increased nitrogen affinity in the heterogeneous surface of the material, leading to trapped nitrogen being unable to desorb [33]. The pore size distribution plot revealed that the main pore sizes of Fe-GFS were in the range of 2–60 nm. BET analysis results showed that the specific surface area of Fe-GFS was 223.45 m^2^/g, pore volume was 0.15 cm^3^/g, and average pore diameter was 2.62 nm. The relatively large specific surface area and abundant pores provided numerous active sites for the adsorption of As(V) and Sb(V).

**Figure 7:**
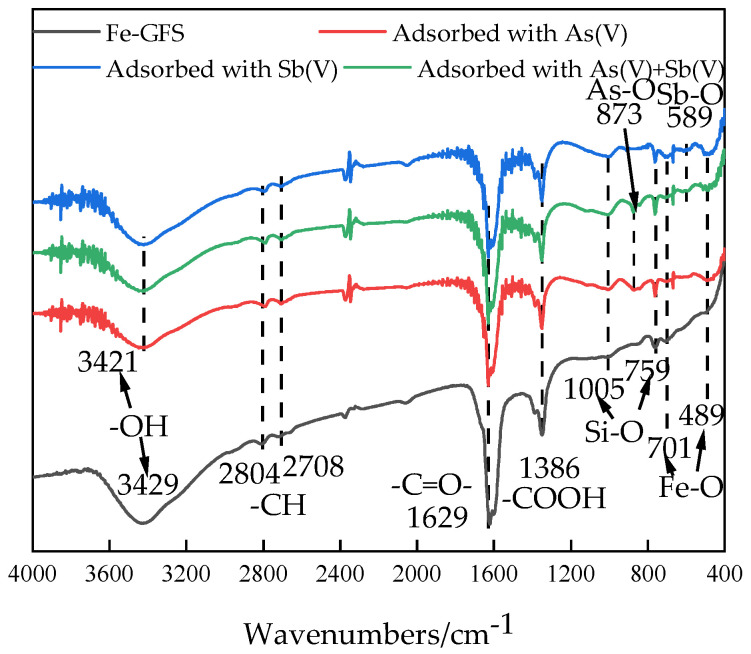
FT-IR spectra before and after Fe-GFS adsorption.

The infrared spectra of Fe-GFS before adsorption and after separate adsorption of As(V) and Sb(V), as well as simultaneous adsorption of As(V) and Sb(V), are shown in Figure 7. The strong and broad absorption peak at 3421 cm^−1^ corresponds to the stretching vibration of -OH. The absorption peaks at 2804 cm^−1^ and 2708 cm^−1^ correspond to the stretching vibration of -CH. The absorption peak at 1629 cm^−1^ corresponds to the stretching vibration of -C=O-. The absorption peak at 1386 cm^−1^ indicates the formation of -COOH [34]. The presence of Si-O is indicated by the absorption peaks at 1005 cm^−1^ and 759 cm^−1^ [29]. The absorption peaks at 701 cm^−1^ and 489 cm^−1^ represent the presence of Fe-O in β-FeOOH [35]. In summary, during the impregnation process of FeCl_3_, β-FeOOH was generated and attached to the surface of the material. The absorption peaks of Fe-GFS at 3421 cm^−1^ decreased after adsorption. Studies show that different arsenic ion forms can be adsorbed on the surface of iron hydroxide and form monodentate complexes with the -OH present on the surface [36]. Regarding Sb(V), which generally exists in the forms of Sb(OH)_3_ and ${\mathrm{Sb}(\mathrm{OH})}_{6}^{-}$ in water, both forms can complex with the -OH on the surface of iron hydroxide to form complexes [37]. Therefore, this may be due to the consumption of the -OH groups on the material surface when forming complexes with arsenate or antimonate ions [38]. After adsorbing Sb(V) and simultaneous adsorption of As(V) and Sb(V), a new absorption peak appeared at 589 cm^−1^, which is caused by the stretching vibration of Sb(V)-O [39]. After adsorbing As(V) and simultaneous adsorption of As(V) and Sb(V), a new absorption peak appeared at 873 cm^−1^, indicating the presence of As-O-Fe [40].

**Figure 8:**
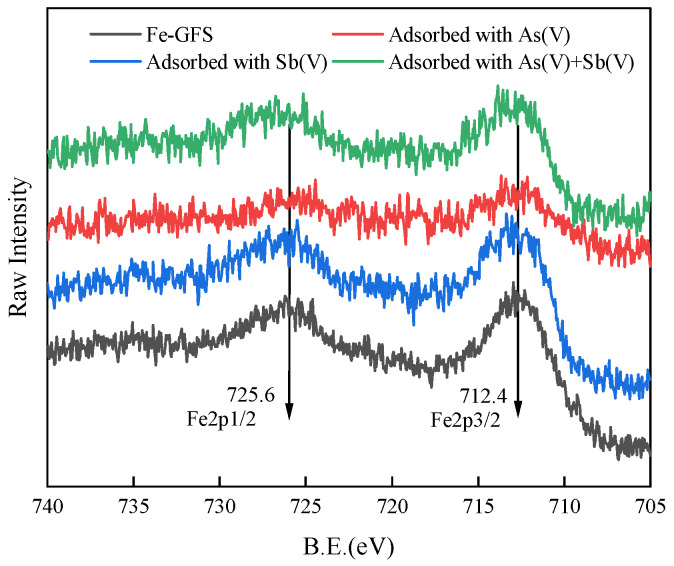
XPS spectra of Fe 2p before and after Fe-GFS adsorption.

**Figure 9:**
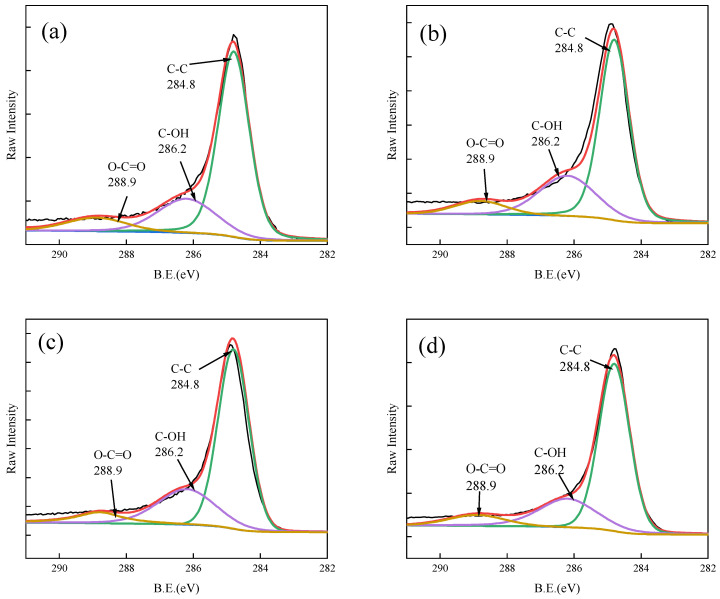
XPS spectra of C 1s before and after adsorption. (**a**) Fe-GFS and Fe-GFS adsorbed with As(V) (**b**), Sb(V) (**c**), and As(V) + Sb(V) (**d**). The red line is the result after fitting.

**Figure 10:**
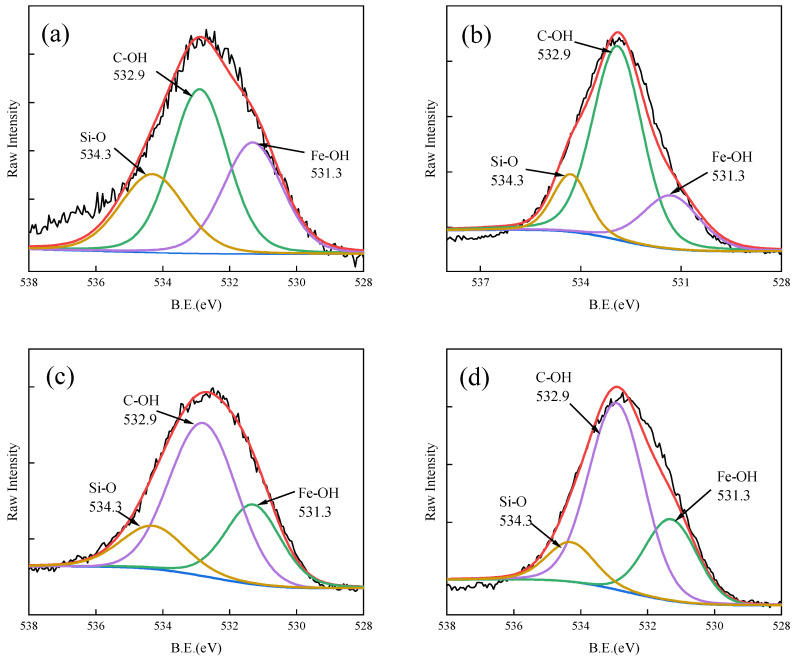
XPS spectra of O 1s before and after adsorption. (**a**) Fe-GFS and Fe-GFS adsorbed with As(V) (**b**), Sb(V) (**c**), and As(V) + Sb(V) (**d**). The red line is the result after fitting.

XPS analysis of Fe-GFS before and after adsorption was conducted. The spectrum analysis results of Fe 2p, C 1s, and O 1s are shown in Figure 8, Figure 9 and Figure 10, respectively. The binding energy peaks at 712.4 eV in the Fe 2p3/2 peak and 725.6 eV in the corresponding Fe 2p1/2 peak represent the peak of FeO(OH) [41]. The O 1s spectrum of Fe-GFS is divided into three peaks at 531.3 eV, 532.9 eV, and 534.3 eV, respectively, indicating the presence of Fe-OH, C-OH, and Si-O groups. Before and after adsorption, the proportion of the Fe-OH group in Fe-GFS decreased significantly: the proportion of Fe-OH before adsorption was 31.43%, decreased to 20.66% after As(V) adsorption, decreased to 26.78% after Sb(V) adsorption, and further decreased to 24.94% after simultaneous adsorption of As(V) and Sb(V). This indicates that during the adsorption of As and Sb, the Fe-OH group decreases, with a greater decrease after As(V) adsorption compared to Sb(V). This suggests that Fe-GFS has a better adsorption effect on As(V) than Sb(V), confirming the adsorption effects of As(V) and Sb(V) in the composite adsorption experiment. This may be because the pKa2 value of H_3_AsO_4_ is 6.96, while the pKa1 value of HSb(OH)_6_ is 2.85. Under neutral pH conditions in the experiment, arsenate anions can retain one or two neutral hydroxyl groups, making it easier to form inner-sphere complexes with the -FeOH on the material surface through ligand exchange [42]. Furthermore, the coordination of Sb(V) with oxygen has a larger spatial structure compared to As(V), resulting in weaker stability of its complexes than arsenic, thus demonstrating a superior adsorption effect on As [16]. The C 1s spectrum of Fe-GFS is divided into three peaks at 284.8, 286.2, and 288.9 eV, indicating the presence of C-C, C-OH, and O-C=O groups. After the adsorption of As(V) and Sb(V), the proportion of O-C=O groups in Fe-GFS also slightly decreased, indicating the consumption of carboxyl groups during the adsorption of As(V) and Sb(V).

## 4. Conclusions

The pH of the solution plays a significant role in determining the protonation strength of the Fe-GFS surface for adsorbing As(V) and Sb(V). As the pH value increases, the adsorption capacity of Fe-GFS for arsenic and antimony decreases notably. Fe-GFS demonstrates effective adsorption of As(V) and Sb(V), with a better adsorption effect observed for As(V) than Sb(V). The maximum adsorption capacities simulated by the Langmuir model were 34.99 mg/g for As(V) and 27.61 mg/g for Sb(V). The Freundlich isothermal adsorption model and the pseudo-second-order kinetic model are found to be suitable for describing the adsorption process of As(V) and Sb(V) by Fe-GFS. The adsorption equilibrium is typically reached within about 200 min. In the binary system of As(V) and Sb(V), the presence of As(V) was observed to consistently inhibit the adsorption of Sb(V) by Fe-GFS. Conversely, low concentrations of Sb(V) were found to inhibit the adsorption of As(V) by Fe-GFS, while high concentrations of Sb(V) promoted the adsorption of As(V) by Fe-GFS. Overall, Fe-GFS primarily adsorbs As(V) and Sb(V) through electrostatic adsorption and surface complexation mechanisms. These findings highlight the importance of pH conditions and the competitive adsorption behavior of arsenic and antimony in aqueous systems when considering the use of Fe-GFS for environmental remediation purposes.

## Figures and Tables

**Table 1:** Fitting parameters of adsorption isotherms of As(V) and Sb(V) on Fe-GFS.

	Langmuir Model			Freundlich Model		
	Q_m_ (mg/g)	K_L_ (L/mg)	R^2^	K_F_ (mg^(1−n)^·L^n^/g)	1/n	R^2^
As(V)	34.99 ± 1.56	0.51 ± 0.10	0.89606	20.47 ± 0.60	0.13 ± 0.011	0.96616
Sb(V)	27.61 ± 2.52	0.055 ± 0.018	0.97158	3.70 ± 0.58	0.42 ± 0.038	0.98406

**Table 2:** Adsorption effects of different adsorbent materials on As(V) and Sb(V).

Material	Adsorption Conditions			Adsorption Capacity (mg/g)		Initial Concentration Range
	T/(°C)	pH	Dose/(g/L)	As(V)	Sb(V)	
Phosphogypsum-modified vinasse biochar [21]	25	7.5	2.5	45.22	8.74	4–90 mg/L
Carbonate rock combined with AMD iron flocs [22]	25	6.0	1.2	20.23	6.78	Sb: 0.608–182.7 mg/L; As: 1.498–299.3 mg/L
Magnetic sludge composite [23]	25	2.6	0.7	18.5	35.7	10–500 mg/L
Fe-GFS	25	3	0.8	34.99	27.61	5–100 mg/L

**Table 3:** Adsorption kinetic parameters of As(V) and Sb(V) on Fe-GFS.

Kinetic Model	Parameters	As(V)	Sb(V)
Pseudo-first-order kinetics	q_e_ (mg/g)	22.53 ± 1.33	18. 80 ± 1.08
	k_1_ (min^−1^)	0.044 ± 0.013	0.017 ± 0.0040
	R^2^	0.8593	0.9078
Pseudo-second-order kinetics	q_e_ (mg/g)	23.77 ± 0.98	20.14 ± 0.91
	k_2_ (g/(mg·min))	0.0031 ± 0.00081	0.0014 ± 0.00034
	R^2^	0.9433	0.9574

**Table 4:** As(V) and Sb(V) adsorbed by Fe-GFS in mixed solutions. Different letters indicate significant differences (*p* < 0.05).

	Concentration/(mg/L)	Adsorption Capacity/mg/g	
		As(V)	Sb(V)
As(V)/Sb(V)	100/0	12.21 ± 0.767 ab	-
	100/10	10.67 ± 0.677 a	0
	100/20	11.29 ± 0.569 a	0
	100/50	13.20 ± 0.448 b	2.20 ± 0.103
	100/100	13.58 ± 1.680 b	3.77 ± 0.227
	0/100	-	14.89 ± 0.952 c
	10/100	1.95 ± 0.108 d	8.74 ± 1.016 b
	20/100	5.04 ± 0.517 c	8.09 ± 1.576 b
	50/100	10.61 ± 1.305 b	6.89 ± 0.237 b
	100/100	13.58 ± 1.680 a	3.77 ± 0.227 a

## Data Availability

The data presented in this study are available upon request from the corresponding author.
